# Reproducibility and Validity of a Food Frequency Questionnaire for Assessing Dietary Consumption via the Dietary Pattern Method in a Chinese Rural Population

**DOI:** 10.1371/journal.pone.0134627

**Published:** 2015-07-31

**Authors:** Xudong Liu, Xiaorong Wang, Sihao Lin, Qingkun Song, Xiangqian Lao, Ignatius Tak-Sun Yu

**Affiliations:** 1 JC School of Public Health and Primary Care, the Chinese University of Hong Kong, Hong Kong SAR, China; 2 Hong Kong Occupational and Environmental Health Academy, Hong Kong SAR, China; 3 Beijing Key Laboratory of Cancer Therapeutic Vaccine, Capital Medical University Cancer Center, Beijing Shijitan Hospital, Beijing, China; Tufts University, UNITED STATES

## Abstract

**Objective:**

This study was conducted to assess the reproducibility and validity of a food frequency questionnaire (FFQ) that was developed to assess the overall dietary consumption via dietary pattern method in a rural population in southwest China.

**Methods:**

A total of 179 participants aged between 40 and 70 years old were included in this study. Participants administered FFQ at baseline (FFQ1) and one year later (FFQ2) to assess the reproducibility. Six 3-day 24-hour recalls (24HRs) were completed between the administrations of two FFQs to determine the validity. Dietary patterns from three separate dietary sources were derived by using principle component factor analysis. Comparisons between dietary pattern scores were made by using Pearson or intraclass correlation coefficient, cross-classification analysis, weighted kappa (κ) statistic and Bland-Altman analysis. The de-attenuated method was adopted to correct the monthly and seasonally variation and the partial correlation analysis was used correct the influence by total energy intake.

**Results:**

Two major dietary factors, labeled as prudent pattern and processed food pattern, were identified. The prudent pattern was characterized by higher factor loadings of wheat, rice, fresh vegetables, bean products, nuts, red meat, white meat and fresh eggs; and the processed food pattern was characterized by higher factor loadings of pickled vegetables, preserved vegetables and salted meat. Between Two FFQs, intraclass correlation coefficients were 0.57 for prudent pattern and 0.55 for processed food pattern, partial Pearson correlation coefficients were 0.51 for the prudent pattern and 0.56 for the processed food pattern; weighted κ statistic ranged from 0.45 (for the prudent pattern) to 0.56 (for the processed food pattern). Between FFQs and 24HRs, de-attenuated correlation coefficients ranged from 0.54 to 0.78 for the prudent pattern and from 0.55 to 0.61 for the processed food pattern; partial Pearson correlation coefficients ranged from 0.41 to 0.56 for the prudent pattern and from 0.42 to 0.44 for the processed food pattern; weighted κ statistic ranged from 0.42 to 0.46 for prudent pattern and from 0.43 to 0.60 for processed food pattern. The Bland-Altman plots and limits of agreement indicated that the deviation/divergence was not obvious for both of the patterns between FFQ1 and FFQ2 and between FFQs and 24HRs.

**Conclusion:**

The study suggests that the FFQ is reasonably reproducible and valid to assess the overall dietary consumption via dietary pattern methods in the Chinese rural population.

## Introduction

Epidemiological studies have suggested that diet and lifestyle are becoming a major issue that influences the population health. A food frequency questionnaire (FFQ) directed to the dietary exposure of conceptual interest in most applications is a comparatively inexpensive and easy-administered approach for assessing long-term dietary habits in large population groups.[1] However, FFQ is sensitive to the diverse lifestyle, eating habits and dietary preferences in the population concerned.[2] Therefore, validation of such an assessment instrument is particularly important, because it may provide valuable insights into the pros and cons of FFQ used in epidemiological studies.[3] Dietary pattern is an integrated and comprehensive method. Dietary patterns can represent the totality of a diet and provide a valuable alternative to measure single nutrients/food intake, and they have been used to explore the diet-disease relationships.[4,5]

In China, more than a half of population has remained to live in rural areas. Increasing attentions should be paid to the health of them. [6] We proposed to conduct a diet-related study in a rural population of southwest China to explore the association between dietary factors and health outcomes. Although some FFQs have been developed to study the dietary intake in China, [3,7–15] no FFQ has been available for the population in rural areas, particularly in southwest China, where different dietary preference and dietary habits existed from other areas.

Thus, we have developed a semi-quantitative FFQ to investigate dietary consumption in this rural area. The present study aimed at assessing the reproducibility and validity of the FFQ in measuring the overall dietary consumption via a comprehensive method—dietary pattern. The reproducibility of FFQ was determined by comparing pattern scores from two FFQs administered with one year apart, and the relative validity of FFQ assessed by comparing pattern scores from FFQs with pattern scores from multiple 24-hour recalls, which served as a standard reference.[1]

## Subjects and Methods

### Subjects

Two hundred participants (aged 40–70 years, men and women) were randomly recruited from Yanting County of Sichuan Province, where over 90% of residents were farmers. The sampling frame for all residents aged 40–70 years was available from local authority. All participants were free of chronic/malignant disease. Out of the invited, 191 (95.5%) participants agreed to take part in this study. During the one-year follow-up, four participants dropped out of the study because of moving to other areas; one participant failed to provide two completed FFQs and six participants did not complete six 3-day 24-hour recalls. Mean ± 3 standardized deviations (SD) of dietary energy intake (calorie) was further used to assess the eligibility of data and those with total energy intake beyond the range of M±3SD would be excluded. One participant was excluded because of reporting an implausible average daily energy intake in FFQ (>8000 kcal per day). Total energy intake of each participant was calculated based on the Chinese food composition tables.[16] Finally, 179 participants were included in the data analysis.

### Study Procedure

**Fig 1:**
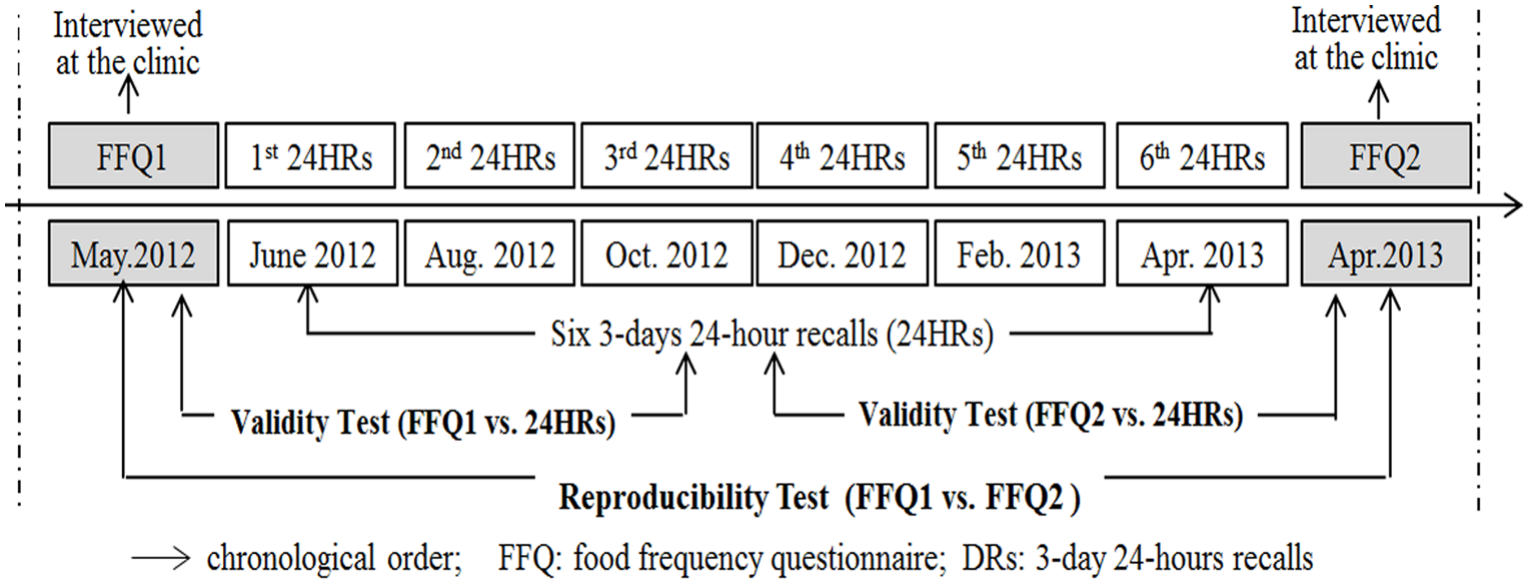
Study design and time frame used in this study. FFQ was administrated at the baseline (FFQ1) and one year later (FFQ2) at rural health clinics by trained interviewers with a face-to face approach. Six times 3-day 24-hour recalls (24HRs) surveys were performed between the two FFQs in every two month. The first 24HRs were performed one month later after the FFQ1. Several interviewers visited to participants with local dialect. All participants were asked to recall all foods (including recipes/ingredients of mixed dishes) and drinks that they consumed from the last day (22:00) to next day (22:00) on the 24-hour dietary recall questionnaires in three consecutive days (including two weekdays and one weekend day). The reliability of FFQ was assessed by comparing the dietary pattern scores between FFQ1 and FFQ2. The Validity of the FFQ was assessed by comparing the dietary pattern scores between FFQs and 24HRs.

The study design with time frame is shown in Fig 1. The study started on 1^st^ May 2012 and ended on 30^th^ April 2013. In collection of dietary data, a standardized tool (a bowl with four levels inside, i.e. ¼, ½, ¾, and 1), a standard portable electronic kitchen scale (EK815J, CAMRY) and an album of food photos [17,18] were provided to the participants. Participants were trained by a registered dietitian about how to estimate the intake frequency and intake amount of each food item. Then, the first FFQ (FFQ1) and the second FFQ (FFQ2) were administered by using a face-to-face interview by trained interviewers with one year apart at the same rural health clinics. Between the two-FFQ interviews, participants completed three 24-hours recalls (24HRs) every two months during a year (a total of 18 recalls in one year). Social-demographic data (age, gender, marital status, educational level, weight and height) were collected with a structured questionnaire when the FFQ1 was conducted.

### FFQ

An FFQ with 76 dietary items was developed to investigate the dietary habits of rural population in southwest China, on the basis of structure of a standardized questionnaire by the National Cancer Institute (NCI tool) [19], but largely modified based on local availability of foods and dietary habits. The procedure of developing FFQ was shown in the supporting information (see Table A in S1 File). The FFQ included twelves groups and 76 items: cereals (9 items) and tuber-crops (2 items), processed vegetables (2 items), fresh vegetables (22 items), fruits (14 items), processed meat (1 item), fresh meat (6 items), fresh eggs (3 item), processed eggs (3 items), bean and products (7 items), nuts and seeds (4 items), alcohol (2 items) and tea intake (1 item) (Table 1). The items listed in the FFQ covered about 98% of commonly consumed diets. In comparison of the NCI tool with our FFQ, there were 32 items existed in both NCI tool and this FFQ (see Table B in S1 File). The other 112 items in the NCI tool did not applied because people in the rural areas rarely or did not consume these foods. Instead, we added 44 dietary items which consumed by local residents to the FFQ. Foods with the intake frequency less than once per two months were not included in the FFQ.

**Table 1:** Food and drinks in the food frequency questionnaire.

Dietary groups		Dietary items in each group
Rice	3	steamed rice (1), rice noodles (2), rice porridge (3)
Wheat	3	noodle (4), steamed wheat bread (5), minor cereals (oatmeal, grits and others) (6)
Corn	3	corn(7), corn bread (8), corn porridge (9)
Tuber crops	2	sweet potato and sweet potato bread (10), potato and products (potato, shredded potatoes, potato chips) (11)
Pickled vegetables	1	pickled vegetables (12)
Preserved vegetables	1	preserved vegetables (13)
Fresh vegetables	22	cabbage (14), Chinese cabbage (15), cauliflower sprouts (16), broccoli (17), green radish (18), spinach (19), pumpkin (20), fresh lettuce leaves (21), carrot (22), egg plants (23), purple cabbage (24), celery (25), cucumber (26), onion (27), sweet peppers (28), bitter gourd (29), green pepper (30), leek (31), watercress (32), chili (33), swamp cabbages (34), tomatoes (35)
Total fruits	14	orange (36), tangerines/mandarin (37), tangelos/pomelo (37), apple (39), pears (40), peach (41), plum (42), apricot (43), loquat (44), cantaloupe (45), watermelon (46), grape (47), persimmon (48), banana (49)
Bean products	7	soy beans (50), fava bean (51), peas (52), fresh tofu (53), soybean sprout (54), dried tofu (55), string beans or green beans (56)
Nuts	4	pumpkin seeds (57), sunflower seeds (58), walnut (59), peanut (60)
Red meat	3	fresh pork (61), fresh beef (62), fresh lamb (63)
White meat	3	duck meat (64), chicken meat (65), goose meat (66)
Salted meat	1	Salted meat (67)
Fresh eggs	3	fresh chicken eggs (68), fresh duck eggs (69), fresh goose eggs (70)
Salted egg	3	Salted duck eggs (71), salted chicken eggs (72), other preserved eggs (73)
Liquor	1	liquor (74)
Beer	1	beer (75)
Tea	1	Tea (76)

There were three questions for collecting information on the consumption of each of the items. First, subjects were asked whether they ever consumed a certain food. If the response was positive, a question asked to indicate how often the consumption was, and how much consumed each time. A food album was attached to the FFQ, including dietary items, measurement tools and weight of each portion size. The weight of each portion size was weighted by a standardized portable electronic kitchen scale (EK815J, CAMRY), which can measure food in gram (g) and drinks in milliliter (ml). The frequency of consumption of food/drink item was categorized into six subgroups, ranging from never to more than twice per day. The intake amount of each food was obtained by multiplying the portion size by weight of portion size. The intake amount of cereals, tuber-crops, vegetables, nuts and meat was measured by using a standardized bowl, which ranged from less than ¼ bowls to more than 3 bowls, with weight of each bowl of food ranging from 100g (vegetables) to 200g (cereals). The intake amount of fruits was measured by nature portions and ranged from less than ¼ fruits to more than 2 fruits. The weight of each fruit with rind being removed ranged from 100g per one (orange) to 150g per one (apple). The intake amount of liquor intake was measued by a cup (1 cup = 50 ml) and ranged from 0 cup (never) to more than 10 cups (500 ml) in 1 cup increment, and beer intake was measured with a bottle (1 bottle = 550 ml) and ranged from 0 bottle (never) to more than four bottles in 0.5 bottle increments. The amount of tea consumption was measured by a electronic scale and ranged from 0g per month to 150g per month in 50g increments.

### Dietary assessment

In the data collection, participants were asked to recall the intake frequency of each item of food/drink and intake amount during the past year. The frequency and amount were recorded by the mid-point value of each category. In summarizing the responses, one month was equal to 4 weeks and one week to 7 days. The responses to the intake frequency were converted into intake times per day. For example, 4–6 times per month was converted to 5 times per month, and then equated to 1.25 (5/4 = 1.25) times per week, and equated to 0.179 (1.25/7 = 0.179) times per day; ≤0.25 bowl per time was converted to 0.125 (0.25/2 = 0.125) bowls per time. Then, the average daily intake of each food (g) or drink (ml) was calculated according to the following formula [Average daily intake = intake frequency (times) per day × intake amount per portion size × weight of the portion size].

For three 24-hour dietary recalls, trained interviewers visited homes of the participants in person with local dialect. The participants were asked to recall all foods (including recipes/ingredients of mixed dishes) and drinks that they consumed from the last day (22:00) to the next day (22:00) with 24-hour dietary recall questionnaire in three consecutive days (including two weekdays and one weekend day). A total of 18 day dietary consumption was collected. 24-hour recall data included single food and mixed dishes. All mixed dishes were converted to a single food. The weight of each food from mixed dishes was calculated according to the ingredients and their portion sizes. For example, a participant recorded that he ate a bowl of fried pork meat with green peppers (1/3 bowl of pork meat and 2/3 bowl of green peppers). This dish was converted into 1/3 bowls of pork meat and 2/3 bowls of green peppers. One bowl of pork meat weighed 200g and one bowl of green peppers weighed 100g. Thus, it is estimated that this dish contained 66.7g pork (200g*1/3) and 66.7g green peppers (100g*2/3). In order to make the dietary daily intake of each food from the 24HRs comparable with that from the FFQs, items from dietary records were separately matched to 76 items listed in the FFQ.

For a convenience of data analysis, the dietary items from FFQs and 24HRs were categorized into eighteen groups, according to local eating habits, dietary guideline and balance diet pagoda for Chinese residents [20], and whether being processed or not. Dietary guideline and balance diet pagoda for Chinese residents provided an ideal dietary pattern from the angle of rational nutrition and balance diet. It divided the food into 10 categories according to the normal eating habits and the characteristics of nutrients provided.[20] In general, dietary consumption in the rural areas lacked of diversity and people in the areas had a preference of consuming processed food (such as pickled and preserved vegetables and salted meat and eggs) and tuber crops.[21] The eighteen groups included pickled vegetables, preserved vegetables, salted meat, salted eggs, rice, wheat, corn, tuber-crops, fresh vegetables, fruits, bean products, nuts, red meat, white meat, fresh eggs, tea, liquor and beer (Table 1).

This study was approved by the Joint Chinese University of Hong Kong—New Territories East Cluster Clinical Research Ethics Committee (The Joint CUHK-NTEC CREC). Informed consent was provided according to the Declaration of Helsinki. All participants completed written consent forms before they joined in the study.

### Statistical analysis

First, average daily intake of each dietary group from the 24HRs was calculated to reduce the variance within-person. The eighteen groups were used across all dietary assessments after their similarities were tested. Paired Wilcoxon rank test was used to compare the intake of each dietary group between different dietary assessments.

Second, principle component factor analyses (PCFA) were conducted with eighteen dietary groups from FFQ1, FFQ2 and 24HRs, respectively. PCFA for each item was first performed separately in men and women (see Table C in S1 File). Final factor scores from overall PCFA (both men and women) were calculated and reported, because similar factors were identified in men and women. Varimax transformation was selected to achieve simple structure with greater interpretability. In determining the number of the factors to retain, eigenvalues (> 1.5), the scree plot and the interpretability of the factors were considered. The Kaiser-Meyer-Olk measure of sampling and Bartlett's test of sphericity were used to determine the relationships between different variables in the factor analysis.[22] The dietary patterns scores were created for each individual by using the regression method. For dietary groups, positive loadings were positively associated and negative loadings were negatively associated with dietary patterns. Higher loadings mean a greater contribution to the dietary pattern. The components with an absolute rotated factor loading of more than 0.4 on a given factor were used to name the factor and are indicated hereafter as ‘dominant components”. The derived factors were labeled on the basis of our interpretation of the data as well as on prior literature.

Third, the reproducibility of the FFQ for assessing the overall dietary consumption was determined by comparing pattern scores from FFQ1 with those from FFQ2 and the validity was assessed by comparing pattern scores from two FFQs with those from 24HRs. Pearson correlation coefficients were used to evaluate the consistency of patterns scores derived from different dietary measurements [23] and partial Pearson correlation coefficients were calculated with adjustment for log10-tranforamtion of total energy intake [24]. De-attenuated Pearson correlation coefficients between FFQs and 24HRs were used to correct monthly and seasonal variation.[25,26] Intraclass correlation coefficient was used to assess the agreement between two FFQs by adjusting for the effect of scale of measures.[27] Cross-classification (via tertile method) analysis was conducted to classify the participants into a same or an opposite tertile and Masson and colleagues criteria [28] was adopted to evaluate the agreement and misclassification. The inter-rater agreement of two assessment methods was analysed by weighted kappa (κ) statistic.[29] A Bland–Altman plot was constructed to visually assess the agreement of dietary pattern scores between different dietary sources.[30] Bland-Altman limits of agreement (LOA) was determined according to the mean agreement (the mean of difference between dietary pattern scores) and 95% LOA (mean ± 1.96 standard deviation of differences) [31].

All statistical analysis was performed using R software (R-3.0.3 for Window) P value less than 0.05 was considered statistically significant. A two-sided test was used in all analyses.

## Results

Among 179 participants, about 59% were males and 95% were married (Table 2). Their mean (S.D.) age was 55 (8.2) years and the mean BMI was 24 (3.4) kg/m^2^. There were 22% and 42% of participants who smoked and drank alcohol, respectively. No significant difference was found in age and BMI between males and females.

**Table 2:** Characteristics of participants in the study.

	Value
Age, years, mean (SD)	54.8 (8.2)
BMI ^a^, kg/m^2^, mean (SD)	23.9 (3.4)
Gender, N (%)	
Male	105 (58.7)
Female	74 (41.3)
Education, N (%)	
Primary school and below	93 (51.9)
Middle school and above	86 (48.1)
Married, N (%)	
Married	170 (95.1)
Divorced and others	9 (4.9)
Smoking status, N (%)	
No	139 (77.7)
Yes	40 (22.3)
Alcohol drinking, N (%)	
No	104 (58.1)
Yes	75 (41.9)
Tea drinking	
No	130 (72.6)
Yes	49 (27.4)

^a^ BMI, body mass index

The median (interquartile) intake of each dietary group from FFQ1, FFQ2 and 24HRs was showed in Table 3. In comparison of the intake of each food group from FFQs with from 24HRs, a significant difference was only found in nuts. When two FFQs were compared, a significant difference was only seen in nuts, white meat and fresh eggs.

**Table 3:** Average daily intakes of eighteen dietary groups from FFQ1, FFQ2 and 24HRs.

Dietary groups	FFQ1 ^a^		FFQ2		6DRs	
	Median	I-Q ^b^	Median	I-Q	Median	I-Q
Pickled vegetables(g)	0.5	2.0	0.5	2.5	0.4	2.7
Preserved vegetables (g)	8.3	9.7	7.8	9.2	8.3	2.1
Salted meat(g)	2.6	6.1	2.7	5.2	2.8	7.2
Salted egg(g)	0.7	2.5	0.8	3.1	0.6	2.3
Rice (g)	122.5	112.5	123.7	78.8	133.0	36.0
Wheat (g)	125.4	45.7	120.3	67.8	129.3	55.3
Corn (g)	38.3	64.3	36.6	58.3	33.6	15.5
Tuber crops (g)	23.4	14.3	24.0	20.4	24.4	27.6
Fresh vegetables (g)	103.4	65.2	90.8	56.3	110.9	36.7
Total fruits (g)	25.1	72.8	26.6	48.8	28.2	17.6
Bean products (g)	23.8	32.3	24.6	25.8	25.5	11.3
Nuts (g)	9.3 ^d^	8.93	8.4 ^c^	7.6	8.5	7.86
Red meat (g)	42.1	40.2	40.5	28.4	43.4	17.7
White meat(g)	2.8 ^d^	7.2	2.9 ^d^	8.6	8.4	9.0
Fresh eggs(g)	18.71	32.1	16.2 ^d^	21.9	19.4	7.7
Tea (g)	0.8	10.0	0.8	15.1	0.9	2.1
Liquor (ml)	9.5	28.6	9.4	19.3	8.6	4.6
Beer (ml)	7.1	66.7	8.6	77.5	8.1	32.9

^a.^FFQ1, the first FFQ administration; FFQ2, the second FFQ administration; 24HRs, six 3-day 24-hour recalls

^b.^Median (Q_50_), I-Q (interquartile) = Q_75—_Q_25_

^c.^Paired Wilcoxon rank test, significantly different from the average of the FFQ1, *P* < 0.05

^d.^Paired Wilcoxon rank test, significantly different from the average of the 24HRs, *P* < 0.05

Two major factors were identified using factor analysis from three dietary sources, respectively (Table 4). The Kaiser-Meyer-Oilskin measure of sampling adequacy was 0.717 for FFQ1, 0.730 for FFQ2 and 0.528 for 24HRs (> 0.5 is regarded as acceptable [22]), and p-values for Bartlett's test of sphericity were all less than 0.001 (<0.001 as acceptable [22]). The first factor, which had a higher factor loading of rice, wheat, total fruits, fresh vegetables, bean products, white meat, red meat, nuts and fresh eggs, was labeled as “prudent pattern.” The second factor, which had a higher factor loading of pickled vegetables, preserved vegetables, salted meat and salted eggs, was labeled as the “processed food pattern.”

**Table 4:** Rotated Factor loadings matrix for two dietary patterns identified from FFQ1, FFQ2 and 24HRs.

Dietary groups	Prudent pattern ^a^ ^b^			Processed food pattern ^a^ ^b^		
	FFQ1	FFQ2	24HRs	FFQ1	FFQ2	24HRs
Pickled Vegetables				0.81	0.82	0.44
Preserved Vegetables				0.74	0.74	0.61
Salted meat			-0.10	0.41	0.78	0.75
Salted egg				0.23	0.29	0.24
Rice	0.74	0.75	0.63	-0.30	0.25	
Wheat	0.42	0.42	0.40			
Corn					0.18	0.18
Tuber crops						0.23
Fresh vegetables	0.86	0.84	0.62			-0.11
Total fruits	0.35	0.30	0.30		0.10	-0.17
Bean products	0.77	0.76	0.77			
Nuts	0.25	0.23	0.13	0.22		-0.43
Red meat	0.58	0.58	0.13			-0.21
White meat	0.87	0.90	0.18			
Fresh eggs	0.49	0.54	0.28		0.23	
Tea	0.11		0.15	0.14		
Liquor			-0.17	0.11		
Beer			-0.14		0.18	0.15
Variance (%)	20.12	19.60	19.35	8.69	11.33	8.75

^a.^FFQ1, the first FFQ administration; FFQ2, the second FFQ administration; 24HRs, six 3-day 24-hour recalls

^b.^Absolute values <0.10 were excluded from the table. For dietary groups, positive loadings are positively associated and negative loadings are negatively associated with the dietary pattern; higher loadings mean a greater contribution to the dietary pattern.

Between FFQ1 and FFQ2, intraclass correlation coefficients were 0.57 for prudent pattern and 0.55 for processed food pattern, crude Pearson correlation coefficients were 0.58 for the prudent pattern and 0.60 for the processed food pattern, and partial Pearson correlation coefficients was 0.51 for prudent pattern and 0.56 for processed food pattern (Table 5). Between FFQs and 24HRs, crude Pearson correlation coefficients ranged from 0.45 to 0.64 for prudent pattern and from 0.46 to 0.50 for processed food pattern, de-attenuated correlation coefficients ranged from 0.54 to 0.78 for the prudent pattern and from 0.55 to 0.61 for the processed food pattern; partial Pearson correlation coefficients ranged from 0.41 to 0.56 for the prudent pattern and from 0.42 to 0.44 for the processed food pattern; weighted κ statistic ranged from 0.42 to 0.46 for prudent pattern and from 0.43 to 0.60 for processed food pattern.

**Table 5:** Correlation coefficients for dietary pattern scores derived from FFQ1, FFQ2 and 24HRs.

Comparison ^a^	Crude PCC ^b^	Partial PCC ^c^	De-attenuated PCC ^d^	ICC ^e^
**Prudent pattern**				
FFQ1 *vs*. FFQ2	0.58	0.52	—	0.57
24HRs *vs*. FFQ1	0.45	0.41	0.54	—
24HRs *vs*. FFQ2	0.64	0.56	0.78	—
**Processed food pattern**				
FFQ1 *vs*. FFQ2	0.60	0.56	—	0.55
24HRs *vs*. FFQ1	0.46	0.42	0.55	—
24HRs *vs*. FFQ2	0.50	0.44	0.61	—

^a.^FFQ1, the first FFQ administration; FFQ2, the second FFQ administration; 24HRs, six 3-day 24-hour recalls.

^b.^Crude PCC, Crude Pearson correlation coefficients, all *P*<0.01

^c.^Partial PCC, Partial Pearson correlation coefficient, adjusted for log10-transformation of total energy intake, all *P*<0.01

^d.^De-attenuated PCC, De-attenuated Pearson correlation coefficients, corrected for the monthly and seasonal variation of food supply, all *P*<0.01.

^e.^ICC, intraclass correlation coefficients, all *P*<0.01

Cross-classification analysis showed that more than 54% of the participants were correctly classified into the same tertile and less than 9% were misclassified into an opposite tertile for both of prudent pattern and processed food pattern when the pattern scores between two FFQs and between FFQs and 24HRs, respectively, were compared (Table 6). Weighed κ statistic depicted a moderate agreement: 0.45 for the prudent pattern and 0.56 for the processed food pattern between two FFQs, 0.42 for the prudent and 0.43 for the processed food pattern between FFQ1 and 24HRs, and 0.46 for the prudent pattern and 0.60 for the processed food pattern between FFQ2 and 24HRs.

**Table 6:** Percentage agreement, kappa statistic and limits of agreement for dietary pattern scores derived from FFQ1, FFQ2 and 24HRs.

Comparison ^a^	Percentage agreement (%)			κ ^b^	Limits of agreement (LOA) ^c^	
	Same tertile	Adjacent tertile	Extreme tertile		Mean agreement (95% LOA)	LOA difference
**Prudent pattern**						
FFQ1 *vs*. FFQ2	56.42	36.31	7.26	0.45	0.04 (-0.88, 0.96)	1.84
24HRs *vs*. FFQ1	54.75	37.99	7.26	0.42	-0.10 (-1.90, 1.70)	3.60
24HRs *vs*. FFQ2	57.54	35.20	7.26	0.46	-0.05 (-1.83, 1.73)	3.55
**Processed food pattern**						
FFQ1 *vs*. FFQ2	64.25	28.49	6.70	0.56	-0.01 (-1.44, 1.46)	2.90
24HRs *vs*. FFQ1	62.01	29.05	8.94	0.43	0.02 (-1.30, 1.34)	2.64
24HRs *vs*. FFQ2	73.18	21.23	5.59	0.60	0.01 (-1.31, 1.33)	2.64

^a.^ FFQ1, the first FFQ administration; FFQ2, the second FFQ administration; 24HRs, six 3-day 24-hour recalls.

^b.^ κ, weighted kappa statistic, all *P*<0.01.

^c.^ Mean agreement, mean of difference between dietary pattern scores; 95% LOA, mean agreement ± 1.96*(standard deviation of difference between measurements); LOA difference, equal to 2.5% LOA-97.5% LOA.

**Fig 2:**
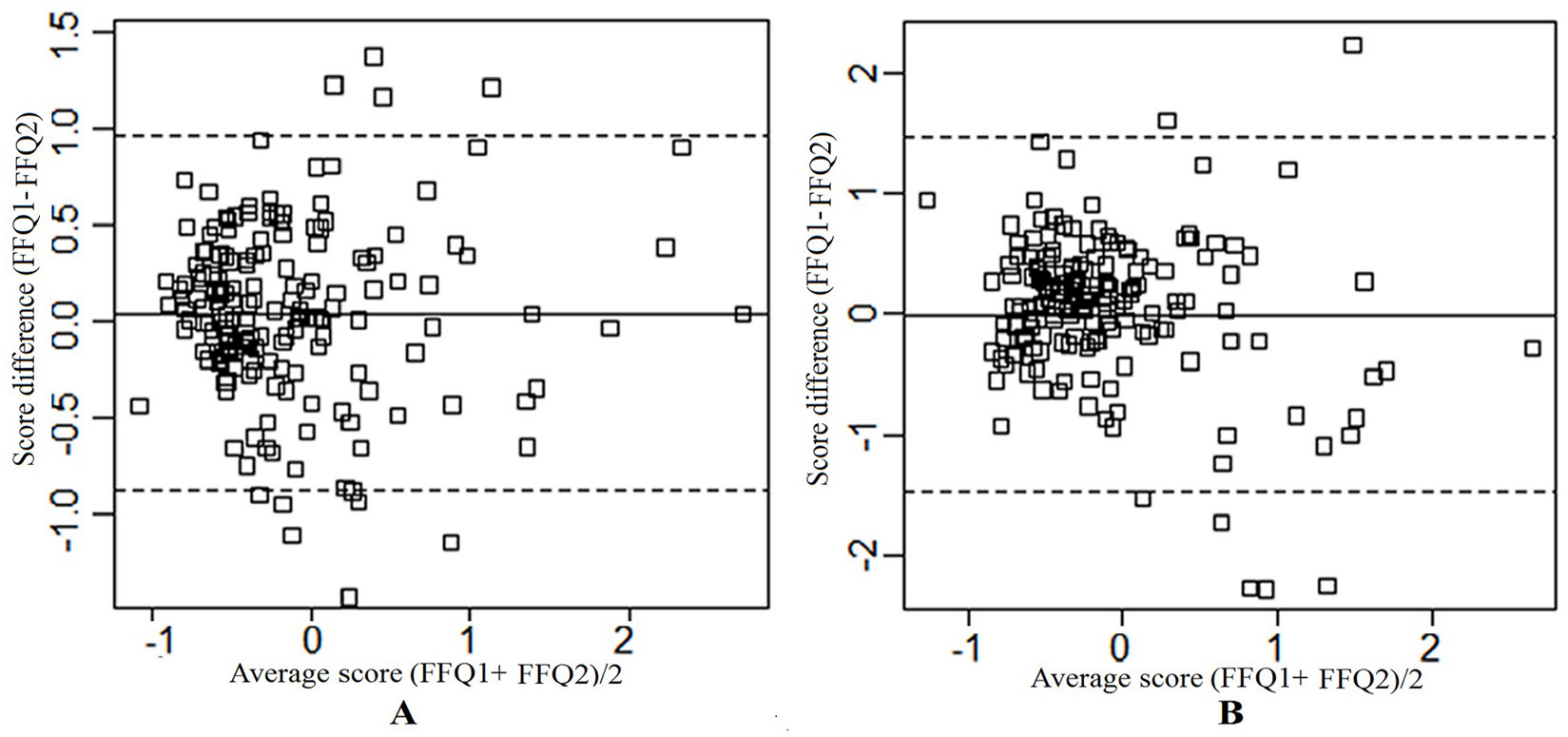
Bland–Altman plots for scores of prudent pattern and processed food pattern derived from FFQ1and FFQ2. A: Prudent pattern; B, Processed food pattern; FFQ1-FFQ2, the patterns score difference of FFQ1 and FFQ2; (FFQ1+FFQ2)/2, average pattern scores of FFQ1 and FFQ2. The solid line represents the mean difference (FFQ1-FFQ2) and the dash lines represent the limits of agreements (mean difference ± 1.96 standard deviations).

**Fig 3:**
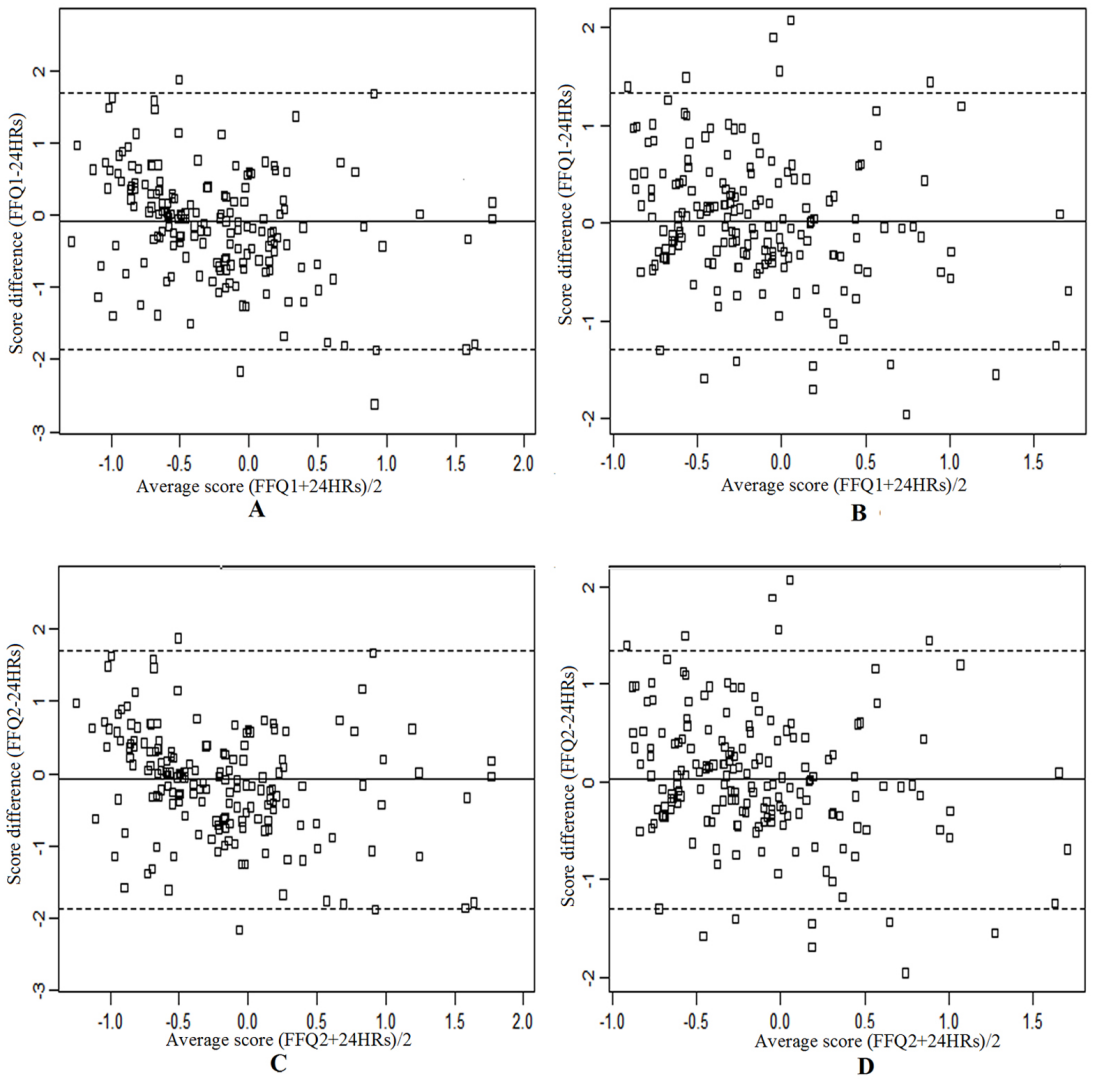
Bland–Altman plots for scores of prudent pattern and processed food pattern derived from two FFQs and 24HRs. A, Prudent pattern (FFQ1 vs. 24HRs); B, Processed food pattern (FFQ1 vs. 24HRs); C, Prudent pattern (FFQ2 vs. 24HRs); D, Processed food pattern (FFQ2 vs. 24HRs); FFQs- 24HRs, the pattern score difference of FFQs and 24HRs; (FFQs + 24HRs)/2, average pattern scores of FFQs and 24HRs. The solid line represents the mean difference (FFQs-24HRs) and the dash lines represent the limits of agreements (mean difference ± 1.96 standard deviations).

In Bland-Altman analysis, the mean of differences of dietary patterns scores were not significant different from zero in all comparisons (Table 6). The mean agreement (95% LOA) was 0.04 (-0.88, 0.96) for prudent pattern and -0.01 (-1.44, 1.46) for processed food pattern between two FFQs, was -0.10 (-1.90, 1.70) for prudent pattern and 0.02 (-1.30, 1.34) for processed food pattern between FFQ1 and 24HRs, and were -0.05 (-1.83, 1.73) for prudent pattern and 0.01 (-1.31, 1.33) for processed food pattern between FFQ2 and 24HRs. Visual inspection of Bland-Altman plots showed that, between two FFQs (Fig 2) and between FFQs and 24HRs (Fig 3), the deviation/divergence was not obvious for prudent pattern and processed food pattern.

## Discussion

In this study, two dietary patterns, i.e., the prudent pattern and the processed food pattern, were obtained by using principle component factor analysis. The components and their factor loadings for the two patterns were consistent in two FFQs. The major components and their factor loadings of the two patterns derived from 24HRs were similar with those from the two FFQs. Yet, there was a small difference, in nuts, salted meat and tea. This might be due to statistic variations and the differences in dietary assessment methods used.[26]

Between FFQs and 24HRs, the de-attenuated correlation coefficients for both two patterns (0.54–0.78) increased after correction of monthly and seasonal variance which indicated moderate to good association. The partial correlation coefficients for both two patterns (0.41–0.56) decreased after adjustment for total energy intake; however, the coefficients were in an accepted range, which further illustrated moderate association between FFQs and 24HRs. Although the methods of comparative validity of dietary patterns were different, the obtained correlations were similar to those reported by other studies, such as the traditional and western patterns in Shanghai’s urban population (0.59–0.64) [13], prudent and western patterns in America (0.45–0.74) [26], Britain (0.35–0.67) [32] and Japan (0.32–0.56) [33], the healthy and western patterns in Swedish (0.41–0.59) [34] and Australian adolescents (0.34–0.47) [24], and healthy and sandwich-drink pattern in Zealand (0.34,0.62) [35] and traditional and western patterns in Iran (0.48–0.75) [36].

When dietary pattern scores for FFQs and 24HRs were classified into tertiles, a higher percentage of participants being classified into the same tertile (>54%) and a low percentage into the opposite tertile (<9%) were shown in both two patterns in this present study, which demonstrated good agreement and lower misclassification between two FFQs and 24HRs, according to the Masson’s criteria [28]. The weighted κ statistic, which overcame agreement by chance, depicted moderate inter-rater agreement (0.42–0.60) for both two patterns. The value of weighted κ statistic of these two patterns were comparable to two dietary patterns reported in Malaysia (0.56 for healthy and 0.75 for less-healthy) [37].

The Bland-Altman method, which estimates the mean agreement and the limits of agreement, is the appropriate one for estimating the absolute validity. The mean agreement, which indicates the mean of difference between dietary pattern scores, was approximately equal to zero for two patterns between FFQs and 24HRs in this study. Though the 95% LOA in prudent pattern were wider than in processed food pattern, these differences were marginal. The 95% LOA for two dietary patterns were acceptable. In comparing with other studies, similar results were also found in Australia adolescent [24] and pregnant women [38]. The Bland–Altman plot, which visually illustrated the degree of agreement between two different measurements [30], further showed that no significant divergence was observed between two FFQs and 24HRs for two patterns.

In addition, for both two patterns, the crude and corrected correlation coefficients, kappa statistics, percentage of agreement and limits of agreement between FFQ1 and 24HRs were all higher/wider than those between FFQ2 and 24HRs, respectively; meanwhile, the misclassification between FFQ1 and 24HRs was higher than those between FFQ2 and 24HRs. This may due to the fact that the 24HRs and FFQ2 reflected the dietary consumption in the same period.

In comparison of two FFQs, intraclass correlation coefficients (0.55–0.57) and partial Pearson correlation coefficients (0.51–0.56) showed moderate to good association for both prudent pattern and processed food pattern. The correlation coefficient in prudent pattern was similar to the prudent pattern in Shanghai (0.54–0.64) [13] and in Iran (0.72, 0.80) [36]. The correlation coefficient in processed food pattern was similar to western patterns in Japan [33], Sweden [34] and America [26]. Cross-classification analysis demonstrated a higher percentage of agreements (>56% classified into the same tertile) and a lower percentage of misclassification (<7.5% classified into the opposite tertile) for two patterns according to Masson’s criteria [28]. The kappa statistic of the two patterns demonstrated moderate inter-rate agreements (0.46–0.56) according to the criteria [29], which were similar to healthy and sandwich-drink patterns (0.57–0.65) reported in New Zealand [35]. Divergence was not obviously shown in Bland-Altman plots for both prudent and processed food patterns between two FFQs. The mean difference was very small for two patterns between two FFQs. Though the 95% LOA in processed food pattern (-1.44, 1.46) was wider than prudent pattern (-0.88, 0.96), both of them were acceptable.

Studies usually derived dietary patterns on the basis of the frequency of consumption rather than the amount of consumption.[34,35] This study took into consideration of both frequency and intake amount each time, which could better reflect real food consumption and dietary habits. The eighteen food groups were comparable among FFQ1, FFQ2 and 24HRs. A significant difference in average daily intake was found only in a few groups, indicating that measurement error of the data from the three sources was not a big concern. The FFQ was administered twice with one year apart, and the 24HRs were conducted within one year. Time intervals used in assessing reproducibility and validity ranged from one month to several years in current existing studies.[24,35,37] For the reproducibility test, a one-year interval is long enough to avoid the problem of learning effects, but the risk of changing dietary habits increases. However, the participants in this study lived in an underdeveloped rural area and had lower-socioeconomic status. Most foods were supplied by the local farmers and daily dietary intake generally lacked diversity.[21] Hence, the risk or chance of dietary habits changing in the one-year interval was small. It is considered that a one-year interval may be appropriate in assessing the validity, [39,40] because the food supply in most rural areas undergoes seasonal variation, and the seasonal and monthly variation can be considered as much as possible within this interval. In this study, the 24-hour recall method was adopted as a reference method to validate the FFQ.[1] Dietary consumption on three consecutive days (including one weekend day and two weekdays) was recorded, so the influence of different diets between weekdays and weekends could be reduced. Furthermore, the dietary recall was conducted every two months during one year. Data from 18 days 24-hour recalls in one year could cover the variability of food consumption during different seasons and reflect the monthly and seasonal variance of dietary intakes.

Major strengths of this study include a high participation rate, detailed data collection by trained interviewers, using different tools, such as food photo album, standardized food scale and standardized container in estimating portion size and intake amount. There are some limitations in this study, however. First, recalling dietary consumption in consecutive days might pose problems of similar intake, as intake in a day might be from leftovers from the previous day. This might influence the estimates of within person variation and have led to an underestimation of the variance ratio. However, it is more suitable to collect multiple 24-hours recalls in consecutive days in this rural setting, because plenty of rural residents lived in remote areas and the level of dietary diversity in rural areas of southwest China was very low. Furthermore, we made efforts to capture a variation between days since that the three days covered two weekdays and one weekend day. Second, the number of study subjects in the study was relative small, which might have let to inadequate study power. Third, the recruited participants aged between 40 and 70 years old, which were the targeted age groups in our forthcoming diets-health outcome study. However, this restricted the application of the FFQ to younger and older adults. In addition, we assumed the dietary patterns derived from the 24-hour recalls as a reference standard. However, 24-hour recalls might also be subject to recall bias, erroneous recording and potential changes in eating behavior,[1] leading to over-estimating or under-estimating food intake. Finally, the two patterns explained about 30% of total variance in three dietary data sources. There were some minor dietary patterns, which were less interpretable and highly variable; therefore they were not presented in the study.

In conclusion, the current data and analysis suggest that the FFQ is reasonably and moderately reproducible and valid in assessing dietary consumption via the dietary pattern method in people living in rural areas of southwest China. The FFQ can be generalized to epidemiological studies in similar areas to study dietary patterns and the relationship to health outcomes.

## Supporting Information

S1 File Supporting information. Procedure of developing the food frequency questionnaire (Table A). Dietary items in the NCI tool and in our food frequency questionnaire (Table B). Rotated factor loadings for two factors in men and female identified from FFQ1, FFQ2 and 24HRs (Table C).
(DOCX)
