# Supplementary material for: Reproducibility and Validity of a Food Frequency Questionnaire for Assessing Dietary Consumption via the Dietary Pattern Method in a Chinese Rural Population
Source: PLoS One. 2015 Jul 31;10(7):e0134627. doi: 10.1371/journal.pone.0134627 (PMC4521698; doi:10.1371/journal.pone.0134627)
Supplement: S1 File — Procedure of developing the food frequency questionnaire (Table A). Dietary items in the NCI tool and in our food frequency questionnaire (Table B). Rotated factor loadings for two factors in men and female identified from FFQ1, FFQ2 and 24HRs (Table C). (DOCX) [file pone.0134627.s001.docx]

**Table A. Procedure of developing the food frequency questionnaire**

| Steps | Activities |
| --- | --- |
| **Step 0**: | Two research epidemiologists translated the NCI tool from English to Chinese. Then one English-Chinese translator, one nutritionist and two other epidemiologists compared the English version with Chinese version. Any unclear or unmatched translation were checked and discussed until there was no discrepancy between Chinese expression and English expression. After that we checked up which item could be applied to the population concerned. |
| **Step 1**: | We reviewed the previous reports in this region, interviewed local residents about the food consumption and dietary habits, collected information of food supply and consumption from local government. Then three members designed the first version of FFQ, respectively. And then the three different FFQs were combined to a full FFQ (the first version of FFQ). |
| **Step 2**: | All members discussed the FFQ that designed by the team members, and obtained the second version of FFQ based on the three different FFQs designed in the step 1. |
| **Step 3**: | We sent FFQ to 2 experts (HCY, WXR) to ask their opinion about the FFQ. Then we revised the FFQ according to the feedback of the experts and obtained the third version of FFQ. |
| **Step 4:** | We asked 10 local residents and let them to see whether the items listed in the second version FFQ can reflect the dietary consumption. Then we revised the FFQ and obtained the fourth version of FFQ |
| **Step 5**: | Then we organized a meeting and invited professionals, local residents and other relative people to take part in the open-discussion. By using the information collected from the discussion, we revised the FFQ and obtained the fifth version FFQ. |
| **Step 6**: | We did a pilot study to verify the FFQ and revised the FFQ according to the information collected (sixth version FFQ). [SQK, ZXQ, XRW, LJ] |
| **Step 7**: | Second round discussion was conducted among the team member, professionals, and local long-term residents. By combining the information, the seventh version FFQ was obtained. |
| **Step 8**: | The eighth version of FFQ was obtained by using the information collected and applied in the study. [SQK, ZXQ, WXR] |
| **Step 9**: | Third round discussion was conducted and the ninth version of the FFQ was obtained. All participants came to an agreement that ninth version of FFQ was finalized version of FFQ. |

**Table B. Dietary items in the NCI tool and in our food frequency questionnaire**

| **NCI dietary history questionnaire (DHQ_NCI)** | | **code 2** | **Our food frequency questionnaire** | |
| --- | --- | --- | --- | --- |
| **code 1** | **items in DHQ_NCI** |  | **items in our FFQ** | **code 3** |
| 1 | tomato/vegetable juice | **1** | / | **/** |
| 2 | orange/grapefruit juice | **2** | / | **/** |
| 3 | other 100 pure/mixed juice | **3** | / | **/** |
| 4 | fruit drinks | **4** | / | **/** |
| 5 | milk | **5** | / | **/** |
| 6 | meal replacement/energy/high-protein beverages | **6** | / | **/** |
| 7 | soft-drink, soda, pop | **7** | / | **/** |
| 8 | beer | **8** | beer | **76** |
| 9 | wine/wine coolers | **9** | / | **/** |
| 10 | liquor/mixed drinks | **10** | liquor | **74** |
| 11 | oatmeal, grits, other cooked cereal | **11** | minor cereals (oatmeal, grits, and others | **6** |
| 12 | cold cereal | **12** | / | **/** |
|  |  | **13** | noodle | **4** |
| / | / | **14** | steamed wheat bread | **5** |
| / | / | **15** | Steamed rice | **1** |
| / | / | **16** | rice noodles | **2** |
|  |  | **17** | rice porridge | **3** |
| 13 | applesauce | **18** | / | **/** |
| 14 | apples | **19** | apples | **39** |
| 15 | pears (fresh, canned, or frozen) | **20** | pears | **40** |
| 16 | bananas | **21** | bananas | **49** |
| 17 | dried fruit, such as prunes or raisins (not including dried apricots) | **22** | / | **/** |
| 18 | peaches, nectarines, or plums? | **23** | peaches | **41** |
| / | / | **24** | plums | **42** |
| / | / | **25** | apricot | **43** |
| / | / | **26** | loquat | **44** |
| / | / | **27** | persimmon | **48** |
| 19 | grapes | **28** | grapes | **47** |
| 20 | cantaloupe | **29** | cantaloupe | **45** |
| 21 | melon, other than cantaloupe (such as watermelon or honeydew) | **30** | watermelon | **46** |
| 22 | Strawberries? | **31** | / | **/** |
| 23 | oranges, | **32** | oranges | **36** |
| 24 | Tangerines or tangelos? | **33** | tangerines/mandarin | **37** |
| / | / | **34** | tangelos/pomelo | **38** |
| 25 | other kinds of fruit? | **35** | / | **/** |
| 26 | COOKED greens (such as spinach, turnip, collard, mustard, chard, or kale) | **36** | / | **/** |
| / | / | **37** | spinach | **19** |
| **/** | / | **38** | pumpkin | **20** |
| **/** | / | **39** | cucumber | **26** |
| **/** | / | **40** | green pepper | **30** |
| **/** | / | **41** | egg plants | **23** |
| **/** | / | **42** | purple cabbage | **24** |
| **/** | / | **43** | bitter gourd | **29** |
| **/** | / | **44** | celery | **25** |
| **/** | / | **45** | leek | **31** |
| **27** | RAW greens (such as spinach, turnip, collard, mustard, chard, or kale) | **46** | swamp cabbages | **34** |
| **28** | coleslaw | **47** | / | **/** |
| **/** | / | **48** | watercress | **32** |
| **/** | / | **49** | Chinese cabbage | **16** |
| **29** | sauerkraut or cabbage (other than coleslaw） | **50** | / | **/** |
| **/** | / | **51** | cabbage | **15** |
| **30** | carrots (fresh, canned, or frozen) | **52** | carrots | **22** |
| **/** | / | **53** | green radish | **18** |
| **31** | string beans or green beans (fresh, canned, or frozen) | **54** | string beans or green beans | **56** |
| **32** | peas (fresh, canned, or frozen) | **55** | peas | **52** |
| **33** | corn | **56** | corn | **7** |
| **34** | broccoli (fresh or frozen) | **57** | broccoli | **17** |
| **35** | Cauliflower or Brussels sprouts (fresh or frozen)? | **58** | cauliflower sprouts | **16** |
| **36** | eat mixed vegetables | **59** | / | **/** |
| **37** | onions | **60** | onions | **27** |
| **38** | vegetables cooked with some sort of fat, including oil spray (not include potatoes) | **61** | / | **/** |
| **39** | some sort of fat, sauce, or dressing added after cooking or at the table (not include potatoes) | **62** | / | **/** |
| **40** | sweet peppers (green, red, or yellow) | **63** | sweet peppers | **28** |
| **41** | fresh tomatoes (including those in salads) | **64** | tomatoes | **35** |
| **42** | lettuce salads (with or without other vegetables) | **65** | fresh lettuce leaves | **21** |
| **43** | salad dressing (including low-fat) on salads | **66** | / | **/** |
| **44** | sweet potatoes or yams | **67** | sweet potato and sweet potato bread | **10** |
| **/** | / | **68** | potato, shredded potatoes, potato chips | **11** |
| **45** | French fries, home fries, hash browned potatoes, or tater tots? | **69** | / | **/** |
| **46** | potato salad | **70** | / | **/** |
| **47** | baked, boiled, or mashed potatoes | **71** | / | **/** |
| **48** | salsa | **72** | / | **/** |
| **49** | catsup | **73** | / | **/** |
| **50** | stuffing, dressing, or dumplings | **74** | / | **/** |
| **51** | chili | **75** | chili | **33** |
| **52** | Mexican foods (such as tacos, tostados, burritos, tamales, fajitas, enchiladas, quesadillas, and chimichangas) | **76** | / | **/** |
| **53** | cooked dried beans (such as baked beans, pintos, kidney, blackeyed peas, lima, lentils, soybeans, or refried beans) (don't include bean soups or chili) | **77** | soy beans | **50** |
| **/** | / | **78** | soybean sprout | **54** |
| **/** | / | **79** | fava bean | **51** |
| **54** | other kinds of vegetables | **80** | / | **/** |
| **55** | rice or other cooked grains (such as bulgur, cracked wheat, or millet) | **81** | / | **/** |
| **56** | pancakes, waffles, or French toast | **82** | / | **/** |
| **57** | Lasagna, stuffed shells, stuffed manicotti, ravioli, or tortellini (not include spaghetti or other pasta.) | **83** | / | **/** |
| **58** | macaroni and cheese | **84** | / | **/** |
| **59** | pasta salad or macaroni salad | **85** | / | **/** |
| **60** | pasta, spaghetti, or other noodles | **86** | / | **/** |
| **61** | bagels or English muffins | **87** | / | **/** |
| **62** | breads or rolls AS PART OF SANDWICHES (including burger and hot dog rolls) | **88** | / | **/** |
| **63** | breads or dinner rolls, NOT AS PART OF SANDWICHES | **89** | / | **/** |
| **64** | jam, jelly, or honey on bagels, muffins, bread, rolls, or crackers | **90** | / | **/** |
| **65** | eat peanut butter or other nut butter | **91** | / | **/** |
| **66** | roast beef or steak IN SANDWICHES | **92** | / | **/** |
| **67** | turkey or chicken COLD CUTS (such as loaf, luncheon meat, turkey ham, turkey salami, or turkey pastrami) | **93** | / | **/** |
| **68** | luncheon or deli-style ham | **94** | / | **/** |
| **69** | Other cold cuts or luncheon meats (such as bologna, salami, corned beef, pastrami, or others, including low-fat) (not include ham, turkey, or chicken cold cuts.) | **95** | / | **/** |
| **70** | canned tuna (including in salads, sandwiches, or casseroles) | **96** | / | **/** |
| **71** | ground chicken or turkey | **97** | chicken meat | **65** |
|  | / | **98** | duck meat | **64** |
|  | / | **99** | goose meat | **66** |
| **72** | beef hamburgers or cheeseburgers | **100** | / | **/** |
| **73** | ground beef in mixtures(such as meatballs, casseroles, chili, or meatloaf) | **101** | / | **/** |
| **74** | hot dogs or frankfurters (not include sausages or vegetarian hot dogs) | **102** | / | **/** |
| **75** | beef mixtures such as beef stew, beef pot pie, beef and noodles, or beef and vegetables | **103** | / | **/** |
| **76** | roast beef or pot roast | **104** | / | **/** |
| **77** | steak (beef) | **105** | fresh beef | **62** |
| **78** | pork or beef spareribs | **106** | fresh pork meat | **61** |
| **/** | / | **107** | fresh lamb | **63** |
| **/** | / | **108** | salted pork meat | **67** |
| **79** | roast turkey, turkey cutlets, or turkey nuggets (including in sandwiches) | **109** | / | **/** |
| **80** | chicken as part of salads, sandwiches, casseroles, stews, or other mixtures | **110** | / | **/** |
| **81** | baked, broiled, roasted, stewed, or fried chicken (including nuggets) | **111** | / | **/** |
| **82** | baked ham or ham steak | **112** | / | **/** |
| **83** | eat pork (including chops, roasts, and in mixed dishes) | **113** | / | **/** |
| **84** | gravy on meat, chicken, potatoes, rice, etc. | **114** | / | **/** |
| **85** | liver (all kinds) or liverwurst | **115** | / | **/** |
| **86** | bacon (including low-fat) | **116** | / | **/** |
| **87** | sausage (including low-fat) | **117** | / | **/** |
| **88** | fish sticks or fried fish (including fried seafood or shellfish) | **118** | / | **/** |
| **89** | fish or seafood that was NOT FRIED (including shellfish) | **119** | / | **/** |
| **90** | oil, butter, margarine, or other fat used to FRY, SAUTE, BASTE, OR MARINATE any meat, poultry, or fish you ate (Please do not include deep frying.) | **120** | / | **/** |
| **91** | tofu, soy burgers, or soy meat-substitutes | **121** | tofu (bean-curd) | **53** |
| **/** | / | **122** | dried tofu | **55** |
|  | / | **123** | pickled vegetables | **12** |
| **/** | / | **124** | preserved vegetables | **13** |
| **/** | / | **125** | corn porridge | **8** |
| **92** | eat soups | **126** | / | **/** |
| **93** | pizza | **127** | / | **/** |
| **94** | crackers | **128** | / | **/** |
| **95** | corn bread or corn muffins | **129** | corn bread | **9** |
| **96** | biscuits | **130** | / | **/** |
| **97** | potato chips, tortilla chips, or corn chips (including low-fat, fat-free, or low-salt) | **131** | / | **/** |
| **98** | popcorn (including low-fat) | **132** | / | **/** |
| **99** | pretzels | **133** | / | **/** |
| **100** | peanuts, walnuts, seeds, or other nuts | **134** | peanuts | **60** |
| **/** | / | **135** | walnuts | **59** |
|  | / | **136** | pumpkin seeds | **57** |
| **/** | / | **137** | sunflower seeds | **58** |
| **/** | / | **138** | / | **/** |
| **101** | energy, high-protein, or breakfast bars such as Power Bars, balance, clif, or others | **139** | / | **/** |
| **102** | yogurt (NOT including frozen yogurt) | **140** | / | **/** |
| **103** | cottage cheese | **141** | / | **/** |
| **104** | cheese (including low-fat; including on cheeseburgers or in sandwiches or subs) | **142** | / | **/** |
| **105** | frozen yogurt, sorbet, or ices (including low-fat or fat-free) | **143** | / | **/** |
| **106** | ice cream, ice cream bars, or sherbet (including low-fat or fat-free) | **144** | / | **/** |
| **107** | cake (including low-fat or fat-free) | **145** | / | **/** |
| **108** | cookies or brownies (including low-fat or fat-free) | **146** | / | **/** |
| **109** | doughnuts, sweet rolls, Danish, or pop-tarts | **147** | / | **/** |
| **110** | sweet muffins or dessert breads (including low-fat or fat-free) | **148** | / | **/** |
| **111** | fruit crisp, cobbler, or strudel | **149** | / | **/** |
| **112** | pie | **150** | / | **/** |
| **113** | chocolate candy | **151** | / | **/** |
| **114** | other candy | **152** | / | **/** |
| **115** | eggs, egg whites, or egg substitutes (NOT counting eggs in baked goods and desserts) | **153** |  |  |
|  |  | **154** | fresh chicken eggs | **68** |
| **/** | / | **155** | fresh duck eggs | **69** |
| **/** | / | **156** | fresh goose eggs | **70** |
| **/** | / | **157** | salted chicken eggs | **72** |
| **/** | / | **158** | salted duck eggs | **71** |
| **/** | / | **159** | preserved chicken eggs | **73** |
| **/** | / | **160** | / | **/** |
| **116** | cups of coffee, caffeinated or decaffeinated | **161** | / | **/** |
| **117** | glasses of ICED tea, caffeinated or decaffeinated, did you drink | **162** | / | **/** |
| **/** | / | **163** | tea | **76** |
| **118** | cups of HOT tea, caffeinated or decaffeinated | **164** | / | **/** |
| **119** | add sugar or honey to your coffee or tea | **165** | / | **/** |
| **120** | add artificial sweetener to your coffee or tea | **166** | / | **/** |
| **121** | non-dairy creamer added to your coffee or tea | **167** | / | **/** |
| **122** | cream or half and half added to your coffee or tea | **168** | / | **/** |
| **123** | milk added to your coffee or tea | **169** | / | **/** |
| **124** | sugar or honey added to foods you ate (do not include sugar in coffee, tea, other beverages, or baked goods) | **170** | / | **/** |
| **125** | margarine | **171** | / | **/** |
| **126** | butte | **172** | / | **/** |
| **127** | mayonnaise | **173** | / | **/** |
| **128** | sour cream | **174** | / | **/** |
| **129** | cream cheese | **175** | / | **/** |
| **130** | salad dressing | **176** | / | **/** |
| **131** | servings of vegetables (not including salad or potatoes | **177** | / | **/** |
| **132** | servings of fruit (not including juices) | **178** | / | **/** |
| **133** | select food eaten at least three | **179** | / | **/** |
| **134** | whether eat any type of vegetarian diet | **180** | / | **/** |
| **135** | whether take any of the following types of fiber or fiber supplements on a regular basis (more than once per week for at least 6 of the last 12 months) | **181** | / | **/** |
| **136** | whether take any multivitamins, such as One-a-Day-, Theragran-,or Centrum-type multivitamins (as pills, liquids, or packets) | **182** | / | **/** |
| **137** | One-a-day-, Theragran-, or Centrum-type multivitamins | **183** | / | **/** |
| **138** | Beta-carotene (NOT as part of a multivitamin) | **184** | / | **/** |
| **139** | Vitamin A (NOT as part of a multivitamin) | **185** | / | **/** |
| **140** | Vitamin C (NOT as part of a multivitamin) | **186** | / | **/** |
| **141** | Vitamin E (NOT as part of a multivitamin) | **187** | / | **/** |
| **142** | take Calcium or Calciumcontaining antacids | **188** | / | **/** |
| **143** | any of the following single supplements you took more than once per week (NOT as part of a multivitamin | **189** | / | **/** |
| **144** | Any of the following herbal or botanical supplements you took more than once per week. | **190** | / | **/** |

**Table C. Rotated Factors loadings for two factors in men and female identified from FFQ1, FFQ2 and 24HRs**

|  | FFQ1 ^a^ | | | |  | FFQ2 | | | |  | 24HRs | | | |
| --- | --- | --- | --- | --- | --- | --- | --- | --- | --- | --- | --- | --- | --- | --- |
| Groups ^b^ | Males ^c^ | | Females | |  | Males | | Females | |  | Males | | Females | |
|  | Factor 1 | Factor 2 | Factor 1 | Factor 2 |  | Factor 1 | Factor 2 | Factor 1 | Factor 2 |  | Factor 1 | Factor 2 | Factor 1 | Factor 2 |
| Pickled Vegetables |  | 0.83 |  | 0.49 |  |  | 0.84 |  | 0.79 |  |  | 0.29 |  | 0.34 |
| Preserved Vegetables |  | 0.77 |  | 0.79 |  | 0.13 | 0.82 |  | 0.75 |  |  | 0.55 | -0.57 | 0.49 |
| Salted meat |  | 0.49 |  | 0.80 |  |  | 0.72 |  | 0.71 |  |  | 0.45 |  | 0.75 |
| Salted egg |  |  |  |  |  | 0.81 |  | 0.87 | -0.16 |  | 0.66 |  |  |  |
| Rice |  | -0.29 |  |  |  |  | 0.40 |  | 0.40 |  |  | -0.26 |  | 0.34 |
| Wheat | 0.37 |  | 0.18 |  |  | 0.44 | 0.20 | 0.20 | 0.11 |  |  | -0.24 |  |  |
| Corn | 0.11 |  |  | 0.10 |  | 0.29 | 0.23 |  | 0.24 |  |  | 0.82 | -0.23 | 0.14 |
| Tuber crops | 0.63 |  | 0.73 |  |  | 0.82 |  | 0.70 |  |  | 0.69 | 0.23 |  | 0.52 |
| Fresh vegetables | 0.45 | 0.10 | 0.47 | 0.10 |  | 0.46 |  | 048 |  |  |  | 0.42 | -0.16 | 0.45 |
| Total fruits | 0.46 | 0.10 | 0.24 | 0.47 |  | 0.44 | 0.12 | 0.23 | 0.19 |  | 0.55 |  | 0.52 |  |
| Bean products | 0.76 |  | 0.72 |  |  | 0.82 |  | 0.85 |  |  | 0.80 | -0.18 | 0.28 | -0.16 |
| Nuts | 0.24 | 0.34 | 0.10 | -0.11 |  | 0.37 | 0.24 | 0.22 | -0.11 |  | 0.11 |  | 0.18 | -0.47 |
| Red meat | 0.64 |  | 0.68 | 0.11 |  | 0.52 |  | 0.55 |  |  | 0.25 |  |  | -0.29 |
| White meat | 0.83 |  | 0.89 |  |  | 0.89 |  | 0.89 |  |  |  |  | 0.80 |  |
| Fresh eggs | 0.51 | 0.13 | 0.31 | -0.38 |  | 0.50 | 0.12 | 0.40 | 0.15 |  | 0.40 | 0.11 | 0.70 |  |
| Tea | 0.16 | 0.17 |  | 0.15 |  | 0.10 | 0.38 |  |  |  |  |  |  | -0.39 |
| Liquor |  |  |  |  |  |  |  |  |  |  | -0.24 | 0.41 |  | -0.17 |
| Beer | -0.11 |  |  |  |  | -0.11 | 0.10 | 0.60 | -0.13 |  | -0.20 | 0.28 |  |  |
| % of Variance | 19.24 | 9.19 | 18.79 | 10.88 |  | 22.17 | 13.40 | 20.83 | 11.40 |  | 12.24 | 8.93 | 10.78 | 9.40 |
| Cumulative % | 19.24 | 38.31 | 18.79 | 29.67 |  | 22.17 | 35.57 | 20.83 | 32.24 |  | 12.24 | 21.18 | 10.78 | 29.78 |
| KMO Measure ^d^ | 0.67 | | 0.63 | |  | 0.68 | | 0.62 | |  | 0.53 | | 0.51 | |
| Bartlett's Test | < 0.001 | | < 0.001 | |  | < 0.001 | | < 0.001 | |  | < 0.001 | | < 0.001 | |

1. FFQ1, the first FFQ administration; FFQ2, the second FFQ administration; 24HRs, six 3-day 24-hour recalls
2. Absolute values <0.10 were excluded from the table. For dietary groups, positive loadings are positively associated and negative loadings are negatively associated with the dietary pattern; higher loadings mean a greater contribution to the dietary pattern.
3. There were 105 males and 75 females.
4. KMO Measure: KMO Measure of Sampling Adequacy; Bartlett's Test : Bartlett's Test of Sphericity
